# A Preliminary Study on Grip-Induced Nerve Damage Caused by a Soft Pneumatic Elastomeric Gripper

**DOI:** 10.3390/polym14204272

**Published:** 2022-10-12

**Authors:** Jin Guo, Jin Huat Low, Vinaya Rajagopal Iyer, Peiyan Wong, Chee Bing Ong, Wen Lin Loh, Chen Hua Yeow

**Affiliations:** 1 School of Life Science, Beijing Institute of Technology, Beijing 100081, China; 2 Department of Biomedical Engineering, National University of Singapore, Singapore 117583, Singapore; 3 Department of Pharmacology, National University of Singapore, Singapore 117600, Singapore; 4 Institute of Molecular and Cell Biology, Agency for Science, Technology and Research, Singapore 138632, Singapore; 5 Department of Surgery, Yong Loo Lin School of Medicine, National University of Singapore, Singapore 119228, Singapore

**Keywords:** soft pneumatic elastomeric gripper, silicone elastomer-based soft pneumatic actuator, sciatic nerve compression, histopathological analysis, rodent free walking analysis

## Abstract

Forceps, clamps, and haemostats are essential surgical tools required for all surgical interventions. While they are widely used to grasp, hold, and manipulate soft tissue, their metallic rigid structure may cause tissue damage due to the potential risk of applying excessive gripping forces. Soft pneumatic surgical grippers fabricated by silicone elastomeric materials with low Young’s modulus may offer a promising solution to minimize this unintentional damage due to their inherent excellent compliance and compressibility. The goal of this work is to evaluate and compare the grip-induced nerve damage caused by the soft pneumatic elastomeric gripper and conventional haemostats during surgical manipulation. Twenty-four Wistar rats (male, seven weeks) are subjected to sciatic nerve compression (right hind limb) using the soft pneumatic elastomer gripper and haemostats. A histopathological analysis is conducted at different time-points (Day 0, Day 3, Day 7 and Day 13) after the nerve compression to examine the morphological tissue changes between the rats in the ‘soft gripper’ group and the ‘haemostats’ group. A free walking analysis is also performed to examine the walking function of the rats after recovery from different time points. Comparing the rigid haemostats and soft gripper groups, there is a visible difference in the degree of axonal vacuolar degeneration between the groups, which could suggest the presence of substantial nerve damage in the ‘haemostats’ group. The rats in the haemostats group exhibited reduced right hind paw pressure and paw size after the nerve compression. It shows that the rats tend not to exert more force on the affected right hind limb in the haemostats group compared to the soft gripper group. In addition, the stance duration was reduced in the injured right hind limb compared to the normal left hind limb in the haemostats group. These observations show that the soft pneumatic surgical gripper made of silicone elastomeric materials might reduce the severity of grip-induced damage by providing a safe compliant grip compared to the conventional haemostats. The soft pneumatic elastomer gripper could complement the current surgical gripping tool in delicate tissue manipulation.

## 1. Introduction

Grasping is an essential action involved in almost all surgical procedures. However, gauging the right grip force required to manipulate tissue safely during surgery remains one of the most challenging skills for young surgeons to master. It is not easy to quantify and define what is the appropriate grip force. Surgeons often require years of training and practice to gain the delicate feel of the tissues to prevent grip-induced damage to the grasped tissue or adjacent structures. Despite the emphasis on the importance of exerting proper grip force during the training, reports showed that more than 50% of errors that occurred in surgical training were associated with uncontrolled excessive force [1,2]. These errors may result in various complications such as pain, pathological scar tissue formation and even irreversible tissue damage that may cause hemorrhage [3]. Two approaches have been proposed to tackle this issue. First, force-feedback systems are incorporated into conventional surgical grasping tools to monitor grip force in real-time [4,5,6,7] and to provide tactile feedback to the surgeons [8,9,10] for safe tissue manipulation. These systems aim to reduce complications caused by grip-induced damage in the surgery. It can potentially quantify the amount of grip force that will cause tissue damage. Surgeons can then adjust the grasping accordingly to prevent unintentional grip damage. Another approach is to fabricate a new type of grasping tools using inherently compliant materials. Soft pneumatic elastomer actuators made of silicone elastomeric materials with low Young’s modulus can perform better in the safe interaction with delicate and fragile objects than the instruments with rigid structures due to their inherent compliance [11,12,13,14,15,16,17,18], which makes them well-suited to grip soft tissues. These deformable materials permit the manipulation of delicate tissues to be achieved by providing safe adaptable gripping.

Conventional surgical grasping tools with sensing capability may allow surgeons to grip, hold and manipulate soft tissue effectively without slippage while maintaining the tool-tissue interaction force at a safe level. Maddahi et al. [2] attached four strain gauges on the prongs of bipolar forceps to analyze force applied to brain tissue in 50 neurosurgical tasks. Sugiyama et al. [4] mounted strain gauge force sensors on the prongs of forceps to measure force profile during neurosurgery. Barrie et al. [5] investigated the manipulation force acting on the abdominal organs of pigs in laparoscopic surgery using load cells integrated at the handle of Johan grasper. Although the tool–tissue interaction force was measured in those studies, it did not represent the actual grasping force at the contact as the sensor had no direct contact with the grasped tissue. Factors, such as friction at the interface and mechanisms within the grasping tool, will affect the accuracy of the force transmission. Recent advancement in flexible electronics and sensing technologies allows the sensors to be placed at the tip of the surgical grasping tools. This can address the aforementioned problems by measuring the direct grasping force at the tool-tissue interface. Qasaimeh et al. [19] developed a polyvinylidene fluoride (PVDF) based tactile sensor and Kim et al. [20] proposed a capacitive-based sensor to measure the grasping force at the surgical instrument tip. However, issues such as space limitations and sterilizability of the sensing system remain challenging for these sensorized grippers to be applied in minimally invasive surgery. In addition, surgeons must constantly monitor the grasping force to minimize tissue damage. This may affect the surgical outcome as the surgeons may not be able to focus fully on performing the surgical tasks since they must remain constantly aware of the grasping force. While some of the sensorized grippers can provide surgeons with force perception, the perception of the feedback force is subjective, such that even the same feedback force may result in different amounts of grasping force applied by different surgeons. This may result in uncontrolled excessive grasping force, leading to severe tissue damage.

On the other hand, new surgical grippers made up of soft silicone elastomeric materials do not require the force-feedback system for delicate tissue manipulation. The grippers of this genre involve very simple design and control to provide safe grasping. Constantly monitoring of grasping force is also not required to prevent tissue damage. These advantages made them more reliable to be used to enhance surgical performance compared to sensorized conventional grasping tools. Hubschman et al. [21,22,23] presented a four-finger micromanipulator for ocular tissue manipulation. It consisted of four silicone-based fingers which could enable a maximum force of 20 mN to displace the retina safely. A soft pneumatic chamber gripper device was designed in [24] to handle delicate tissue during surgery. The elastomeric gripping component generated a grasping force of three times smaller than the force of forceps, and yet it can be used to pick up and hold objects up to 2 mm like the forceps did. Rateni et al. [25] developed a soft robotic gripper fully made up of elastomeric material for safe tool-tissue interaction in surgery. The generated grasping force of 1 N was within the acceptable range of manipulation force for tissue manipulation in minimally invasive surgery.

Despite various soft elastomeric grippers have been proposed for safe surgical manipulation, medical applications of these grippers are not well explored as in-vivo tests are not performed to access the grip performance in surgery. The use of animal models is important to examine the tool-tissue interaction in the environment that is closer to real surgical scenario. In addition, evaluation of the tissue recovery from grip-induced damage, an important aspect to determine the performance of a surgical grasping tool, is lacking in those studies. In this study, sciatic nerve compression on the right hind limb of rats was conducted to evaluate the grip performance of the soft pneumatic surgical gripper proposed in our previous work for digital nerve repair surgery [26]. The gripper consists of a soft pneumatic silicone elastomer actuator encased in a rigid casing. The actuator inflates and provides compliant grip to the tissue when the pressurized air is supplied to the pneumatic air channel. Sciatic nerve compression is performed as the sciatic nerve is the most frequently used model for studies on nerve regeneration and recovery after peripheral nerve injuries [27,28]. These injuries are common and debilitating, affecting 13–23 per 100,000 persons annually and the severe cases will eventually lead to long-term functional disability [29]. The repair surgery remains a major challenge for surgeons and factors such as improper soft tissue handling, unintentional damage to the adjacent structures, etc., result in a complication rate of 2.91% after surgery [30].

Histopathological analysis was conducted at different time points to access the tissue recovery from grip-induced damage introduced by the soft pneumatic elastomeric gripper compared to the conventional steel haemostats. Free walking behavioral tests were also conducted to evaluate the functional recovery of the sciatic nerve at different time points. The aim of this study is to show that the silicone elastomer-based soft pneumatic gripper can be safely used to grip, hold, and manipulate tissue and eventually reduce the risk of complications caused by unintentional grip-induced damage in surgery. This is the first study to investigate tissue recovery after grip-induced damage over two weeks using rodent models.

In this paper, first, we introduced the soft robotic surgical gripper system and surgical procedures involved in sciatic nerve compression. Next, we described the evaluation tests used to assess the nerve damage, such as histopathological analysis and functional assessment of limb recovery. Finally, we discussed and compared the grip-induced nerve damage caused by the soft pneumatic elastomeric gripper and conventional haemostats.

## 2. Materials and Methods

### 2.1. Soft Hybrid Surgical Gripper

**Figure 1:**
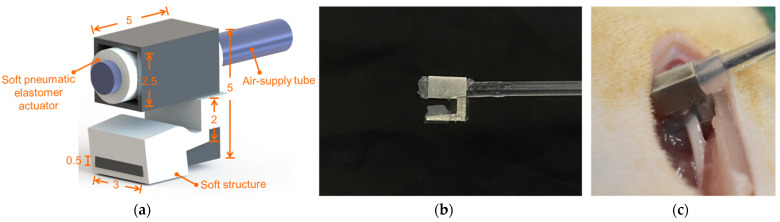
The soft hybrid surgical gripper used in this study: (**a**) The dimension of the soft hybrid surgical gripper in millimeters; (**b**) The soft hybrid surgical gripper before gripping; (**c**) Gripping the rat sciatic nerve using the soft hybrid surgical gripper.

The design and functionality of the two-arms soft robotic surgical gripper system have been evaluated in our previous work [26]. Each arm consists of a soft hybrid surgical gripper, a pluggable connector and three variable-stiffness ball joints with a soft pneumatic locking actuator in each ball joint. The soft pneumatic elastomer actuator, positioned inside the hook-shaped gripper shell, is inflated to provide compliant grip and soft contact with nerve tissue (illustrated in Figure 1a,b). Upon pressurization of the pneumatic channel, the soft pneumatic gripping actuator inflates to compress and push the nerve against the rigid hook shell to hold the nerve in place. The inflated soft pneumatic gripping actuators are capable of not only providing steady gripping force when the supplied air pressure is constantly kept, but also absorbing much of the energy arising from a collision. Thus, the soft pneumatic gripping actuators allow the soft hybrid surgical grippers to effectively avoid unintended excessive stress damage to nerve tissues while handling the nerves using the conventional haemostats requires extreme caution. The soft pneumatic locking actuators inflate to lock the ball joints and hence secure the arm in place when the pressurized air is supplied to the air channel. Ecoflex 0030 (Smooth-On Inc., Macungie, PA, USA, 1:1 parts A:B), a type of platinum-cure silicone elastomer with a durometer hardness of Shore 00-30, was used to fabricate the soft pneumatic gripping actuators. The soft pneumatic elastomer actuators made of Ecoflex 0030 is capable of withstanding high temperatures up to 232 degrees Celsius [31,32], which allows the soft hybrid surgical grippers to be sterilized through high temperatures. Additionally, the soft pneumatic gripping actuator was designed as a pluggable component in the soft hybrid surgical gripper. The soft pneumatic gripping actuator is low-cost and designed for one-time usage, which can be thrown away after the surgeries. Three pedal switches were used to control the inflation and deflation of the soft pneumatic elastomer actuators. Compared to our previous gripper design, the hook structure of the soft hybrid surgical gripper used in this study was enlarged and improved by covering a wave-shaped soft structure also made of Ecoflex 0030. The nerve can be enclosed by the inflated soft pneumatic gripping actuator and the soft structure during gripping operations (illustrated in Figure 1b).

### 2.2. Rodents

Twenty-four seven-week-old male Wistar rats, weighing 250–300 g (InVivos Pte Ltd., Singapore), were used in this study. The study was conducted in accordance with the approval (R17-1414) from the Institutional Animal Care and Use Committee (IACUC) of National University of Singapore. The rats were housed in individually ventilated cages at 19 °C to 22 °C with a 12-h light/dark cycle. Teklad global 18% protein rodent diets (Envigo, Huntingdon, UK) and water were available ad libitum. All rats were weighed prior to the experiment being conducted at each time point. The rats will be excluded from the study in cases of disease, infection, suffering, or pathologies not directly connected to the procedure.

### 2.3. Surgical Procedures

**Figure 2:**
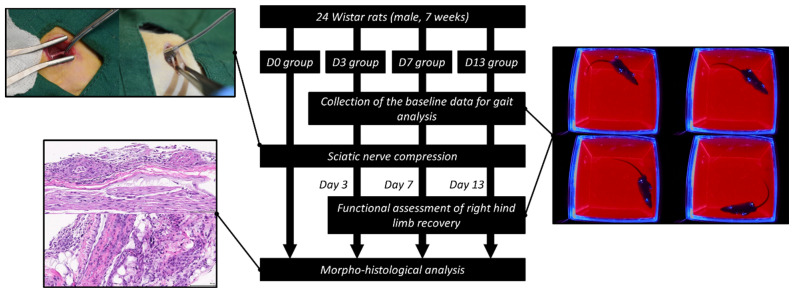
Overview of the experimental protocol.

The overview of the experimental protocol was illustrated in Figure 2. Twenty-four Wistar rats were first divided into four different subgroups according to the different time points (D0, D3, D7, and D13) respectively, with six rats in each group. In each group, the six rats were further divided into two subgroups, including the ‘soft gripper’ subgroup and the ‘haemostats’ subgroup, with three rats in each subgroup. The baseline data for gait analysis of the rats in D3, D7, and D13 groups were collected prior to the surgery.

The haemostats and the soft hybrid surgical gripper were sterilized through uperization at the temperature of approximately 200 degrees Celsius for 20 min before being used by the surgeon. During surgical procedures, the rats were tranquilized with 5% isoflurane then general anaesthesia was induced with ketamine-xylazine (100 mg/kg + 10 mg/kg). Maintenance of general anaesthesia was achieved with 1–2% isoflurane in 100% oxygen delivered via nose cone. Pre-emptive analgesia using buprenorphine hydrochloride (Ilium Temvet) 0.05 mg/kg subcutaneously, and antimicrobial prophylaxis with enrofloxacin (Bayer, Leverkusen, Germany) 10 mg/kg subcutaneously were also provided. Fur was clipped from the lateral surface of the right hind limb from the iliac crest to the mid-tibia. The skin was disinfected with povidone iodine and 70% ethanol. Surgical procedures were performed with strict aseptic techniques.

**Figure 3:**
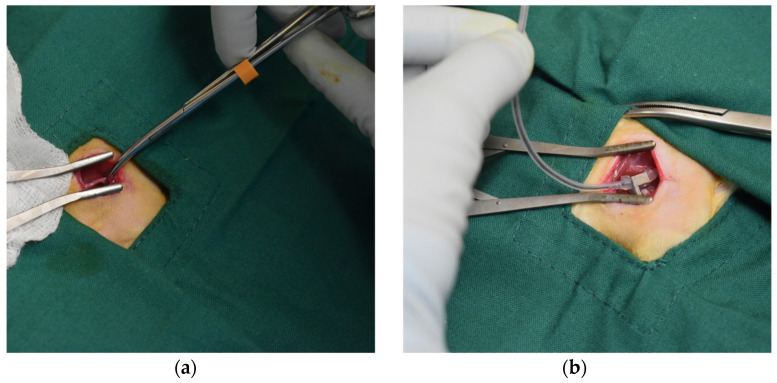
Sciatic nerve compression on the right hind limb was conducted with (**a**) the conventional steel haemostats and (**b**) the soft robotic surgical gripper device.

Surgical access to the sciatic nerve was achieved via a 2 cm longitudinal skin incision on the lateral thigh parallel to the femur. Blunt dissection of the biceps femoris revealed the sciatic nerve. Sciatic nerve compression on the right hind limb was conducted with the soft hybrid surgical gripper and conventional steel haemostats (illustrated in Figure 3). The rats were randomly divided into two subgroups (‘soft gripper’ group and ‘haemostats’ group), with six rats in each group. For the ‘haemostats’ group, the nerve was held with the tips of the instrument using the minimal force necessary to keep the tips apposed in contact, to simulate gentle nerve handling. For the ‘soft gripper’ group, the sciatic nerve was gripped using the inflated soft pneumatic gripping actuator at the air pressure of 150 kPa, which was proven to be able to complete the nerve repair surgery in our previous work. We also estimated the relationship between the input air pressure and the contact force with respect to different positions within the hook retractor of the hybrid soft surgical gripper based on a sensitive force sensor [26]. The compression using the haemostats and the soft gripper lasted the same amount of time. After the compression, the biceps femoris was apposed using 5/0 glycolide/lactide absorbable suture (Polysorb, Medtronic, Minneapolis, MN, USA) in a simple continuous pattern and the skin was also closed with 5/0 glycolide/lactide suture in a buried subcuticular continuous pattern.

For the D0 subgroup, the sciatic nerve specimens were collected for post-mortem histopathological analysis after the rats were euthanized. Free walking behavioral tests were performed on the rats at 3, 7, and 13 days respectively after the surgery to examine the functional recovery of the limb. After the tests, the rats were euthanized, and the sciatic nerve specimens were harvested for the evaluation of nerve damage based on histology and histopathological analysis.

### 2.4. Gait Analysis

**Figure 4:**
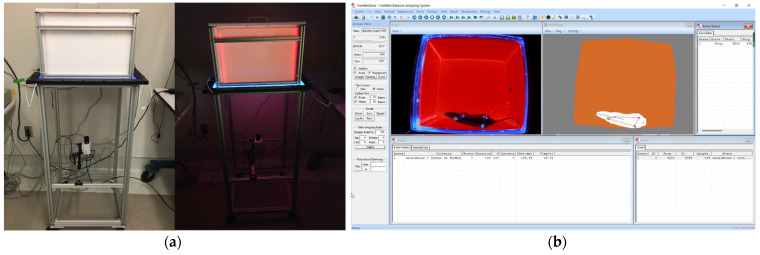
The devices and software used for gait analysis in this study: (**a**) Free Walk Box; (**b**) FreeWalkScan software.

Free walking behavior was used as a measure to examine the functional recovery after sciatic nerve compression. Animals were allowed to move freely in an open field box with a glass floor [33] (Free Walk Box, CleverSys, Reston, VA, USA) for 10 min (shown in Figure 4a). The apparatus was capable of providing a novel and innovative approach for collecting data for gait analysis. The dimensions of the box were 40 cm (width) × 40 cm (length) × 30 cm (height), and adjustable light panels were used to distribute light uniformly over the entire glass floor to enable the detection of the paws. A low-intensity red background light was used to separate the image of the body from the brightly lit paws. The videos of the ventral view were captured by a CCTV camera (Panasonic WV-CP304) and analyzed using FreeWalkScan software (Cleversys, Reston, VA, USA), which used algorithms to reliably define the movement of the animal and paw area in contact with the glass floor, as illustrated in Figure 4b. Various gait parameters, such as paw pressure, foot contact size, stride, stance duration, swing duration, and overlap region, were determined from the software. Events that occurred when the animal was sitting in an area and not moving were not included. Pre-operative evaluation was performed on all the rats prior to the surgery to collect the baseline data. Post-operative evaluations at different time points after the surgery were conducted to evaluate the recovery of the nerve.

Within each timepoint, a ratio for each of the gait parameters was obtained by [Right limb (compression)]/[Left limb (non-compression)]. Post-surgical fold change was then obtained by {[Post-surgical ratio] − [Pre-surgical ratio]}/[Pre-surgical ratio]. Means and standard error of means were shown and analyzed. Due to the small numbers of animals per group, statistical tests were not run. Rather, trends as observed from the graphical visualizations were described.

### 2.5. Histology

**Figure 5:**
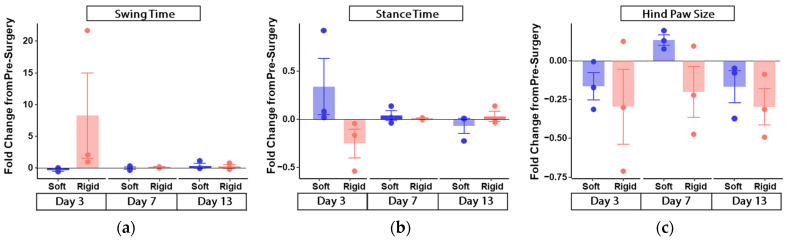

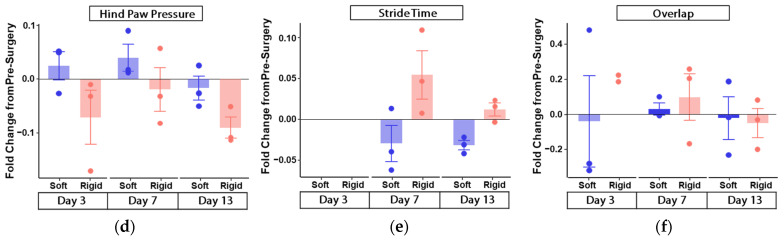
The results of gait analysis parameters across different time-points: (**a**) Swing time; (**b**) Stance time; (**c**) Hind paw size; (**d**) Hind paw pressure; (**e**) Stride time; and (**f**) Overlap.

**Figure 6:**
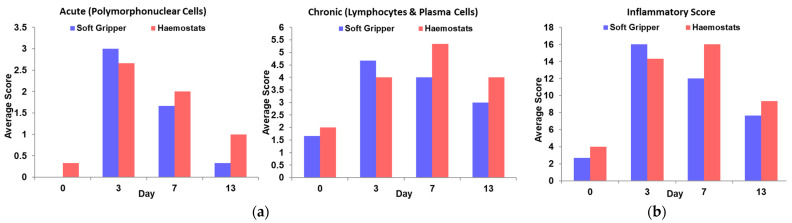
The average score of (**a**) acute, chronic and (**b**) overall inflammation from the histological analysis.

The harvested nerve specimens were immediately immersed in 10% neutral buffered formalin solution, and fixed for 48 h. The tissues were placed into cassettes and processed with Sakura VIP Tissue Processor, through increasing alcohol concentrations and finally xylene and paraffin. After processing, tissues were embedded into a paraffin block and sectioned with rotary microtome into 5 µm thick sections. The slides with section were dried and placed into 60 degrees incubator for about 15 min before H&E staining with Leica Autostainer XL. Longitudinally sectioned of the nerve specimens, with visualization of the two test sites on each specimen were generated, thereafter evaluated and graded semi-quantitatively by a board-certified pathologist (CBO) for cellular response and tissue response. One of the most commonly used grading systems [34] was employed in this study, in which the nerve damage is based on a 0–5 scale, including 0-NAD (no abnormalities detected), G1 (minimal), G2 (mild), G3 (moderate), G4 (marked), and G5 (severe). Degrees of inflammatory cell infiltrate of polymorphonuclear cells, macrophages, lymphocytes, plasma cells, and mast cells were evaluated under cellular response. Factors of tissue response evaluated include axonal degeneration, vascular congestion, hemorrhage, fibrosis, and angiogenesis. The average scores based on the grading system were used to evaluate the damage caused by different grippers (See Figure 5 and Figure 6).

## 3. Results and Discussion

### 3.1. Gait Analysis

Rats tend to walk with symmetric gaits, with equidistantly spaced left and right limb foot-strikes occurring at 50% of their gait cycles [35]. In congruence, we observed that the ratio of the measured parameters for the right to left hind limbs at pre-surgery was close to 1. Post-surgery, we would expect the unilateral injury to result in changes from a symmetric to asymmetric gait. As such, changes to the ratio, of right to left hind limbs, pre- and post-surgery can be used to identify abnormal gait associated with pain due to the induced unilateral injury. Gait parameters of hind limb gait in both gripper groups are presented in Table 1.

From the experimental results shown in Figure 5, we noted that the largest differences between rigid and soft gripper, and between pre- and post-surgery were observed mostly at post-surgery day 3, especially for swing and stance time (see Figure 5a,b). At post-surgical days 7 and day 13, the soft and rigid gripper groups looked similar, and with fold change of post- to pre-surgery being close to 1. Stance duration has been used as an indicator to evaluate motor nerve recovery [36]. Various research studies reported that stance duration was reduced significantly in the injured limb and the shortened duration was related to nerve dysfunction and decreased power in the compromised limb [37,38]. Therefore, the increased stance duration in the soft gripper group indicated less severe grip-induced damage on the rats’ sciatic nerve from the soft gripper or better recovery of the damaged nerve compared to the rats in the haemostats group.

We observed that for hind paw size and hind paw pressure (shown in Figure 5c and 5d respective), the rigid gripper group showed a negative fold change from pre-surgery and more negative than the soft gripper group, with post-surgery ratios being smaller than that of 1. This suggested that for the size and pressure, the compression right hind paw of the rigid gripper group was smaller than that of the non-compression left hind paw. Hence, the compression injury from the rigid gripper may have caused the rats to shift their weight from the affected limb to the contralateral limb to protect the injured limb in the haemostats group. These observations may indicate the changes in limb loading due to pain evoked by movement. With paw size was reduced further in the haemostats group and this may indicate that this group of rats experienced more pain compared to the rats from the soft gripper group. As sciatic nerve injury is associated with neuropathic pain [39], a reduction in pain experienced by rats from the soft gripper group indicated reduced sciatic nerve injury. Thus, these findings demonstrated that the soft hybrid surgical gripper may minimize grip-induced damage by providing a safe compliant grip compared to the conventional haemostats.

We were not able to record accurate measures of stride time at post-surgery day 3, as such, have only shown the data for days 7 and 13. Here, we observed that the soft gripper group showed a negative fold-change from pre-surgery, whereas the rigid gripper group showed a positive change from pre-surgery (illustrated in Figure 5e) at both days 7 and 13 post-surgery. We did not observe much difference between pre-surgery and post-surgery overlap across both groups (illustrated in Figure 5f).

### 3.2. Histology

From the experimental results, the extent of acute and chronic inflammatory cell infiltrate generally appeared less severe in the ‘soft gripper’ group than the ‘haemostats’ group, as observed by the relatively lower average acute and chronic scores on Day 0, 7 and 13 (shown in Figure 6a). Moreover, it was important to note that the ‘haemostats’ group exhibited acute inflammatory response at Day 0, but not for the ‘soft gripper’ group. Although both the groups had similar types of inflammatory cells present, the extent of inflammation in the ‘soft gripper’ group seemed to peak earlier at Day 3 and dwindle thereafter, while the extent of inflammation in the ‘haemostats’ group seemed to persist from Day 3 onwards with relatively higher inflammatory scores than the ‘soft gripper’ group at Day 7 and 13 (illustrated in Figure 6b).

**Figure 7:**
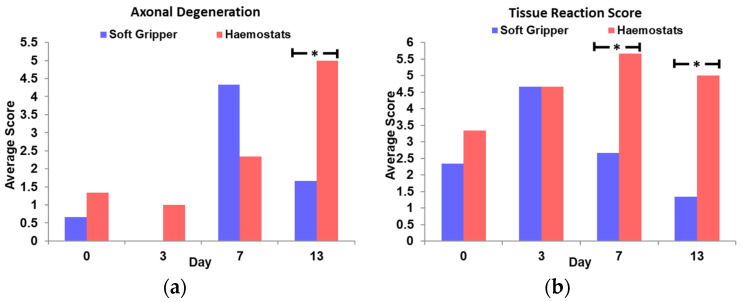
The average score of (**a**) axonal degeneration and (**b**) tissue reaction score across different time-points (* means *p* < 0.05).

In addition, the degree of axon degeneration was found to be significantly greater (*p* < 0.05) in the ‘haemostats’ group than the ‘soft gripper’ group at Day 13 (illustrated in Figure 7a). Moreover, the extent of tissue reaction was significantly higher (*p* < 0.05) in the ‘haemostats’ group than the ‘soft gripper’ group at both Day 7 and 13 (shown in Figure 7b).

**Figure 8:**
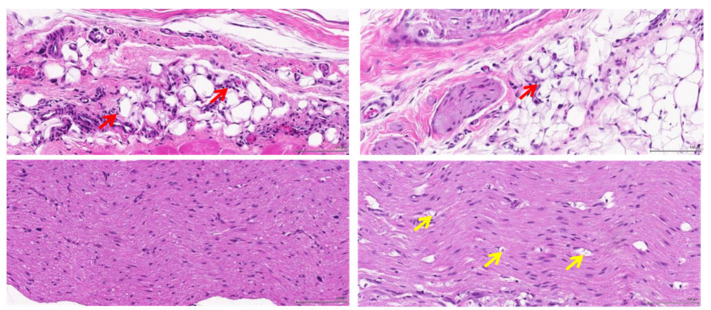

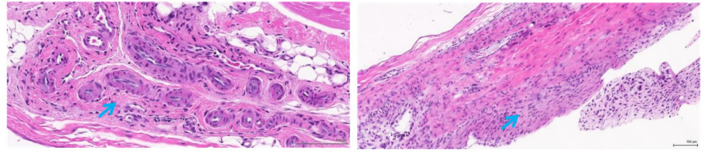
There is mild inflammation and fibrosis present around the nerve fibres at the grip sites by the soft gripper: (Histopathological findings at D13, 20×, H&E, Inflammatory cell infiltrate (red arrow), Axonal degeneration (yellow arrow), Fibrosis (blue arrow)).

**Figure 9:**
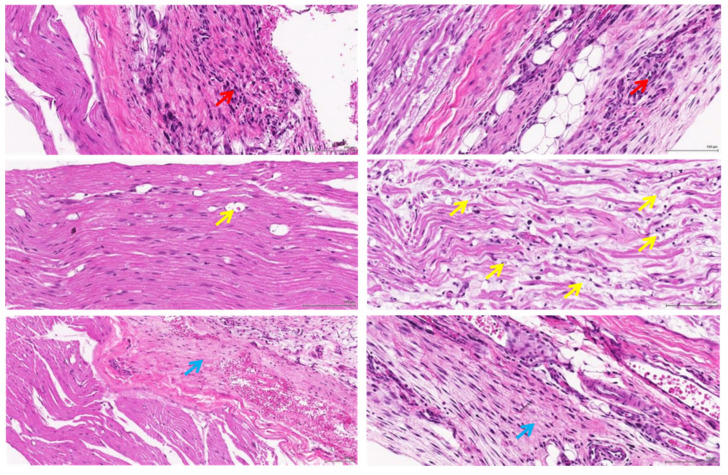
There is moderate inflammation and fibrosis present and mild axonal degeneration at the grip sites by the haemostats: (Histopathological findings at D13, 20×, H&E, Inflammatory cell infiltrate (red arrow), Axonal degeneration (yellow arrow), Fibrosis (blue arrow)).

Based on the histopathological findings, while inflammation and fibrosis were variably present among samples and often focused on suture sites (foreign-body reaction), there appeared to be a visible difference in the degree of axonal vacuolar degeneration between the groups, which could suggest the presence of substantial nerve damage in the ‘haemostats’ group. Figure 8 and Figure 9 presented the mild and moderate inflammation and fibrosis caused by the ‘soft gripper’ and ‘haemostats’ groups at D13.

## 4. Conclusions

In this paper, we preliminarily explored the grip-induced damage caused by a soft hybrid surgical gripper. The grip performance of the soft hybrid surgical gripper was evaluated by histopathological analysis and by investigating nerve recovery after grip-induced damage over two weeks using rodent models. The results showed that the soft gripper caused less severe damage and the rats in the soft gripper group exhibited larger paw pressure and stance duration in the affected right hind limb. From the results of the comparative experiments with the conventional haemostats, the soft hybrid surgical gripper can be implemented as a gripping solution to minimize unintentional grip-induced damage during surgery due to its unique compliance and adaptability characteristics. Therefore, the soft robotic surgical gripper could complement the current surgical gripping tool in delicate tissue manipulation. In our future work, we will further improve the design of the soft pneumatic elastomer gripper to reduce its end volume and expand its medical applications. In addition, histological analysis and gait analysis based on the new gripper design will also be conducted to examine the nerve damage and recovery.

## Figures and Tables

**Table 1:** Gait parameters of hind limb gait in soft gripper group and haemostats group. Data are expressed as mean post-surgical fold change (injured right hind limb vs normal left hind limb, SEM: standard error of the mean).

Post-Surgical Day	Gripper	Size	SEM	Pressure	SEM	Stance	SEM	Swing	SEM	Overlap	SEM	Stride	SEM
D3	Soft	−0.164	0.089	0.025	0.026	0.339	0.291	−0.386	0.167	−0.039	0.260		
	Rigid	−0.297	0.241	−0.071	0.050	−0.252	0.149	8.234	6.718	0.205	0.015		
D7	Soft	0.132	0.034	0.040	0.025	0.038	0.052	−0.056	0.174	0.031	0.035	−0.030	0.022
	Rigid	−0.202	0.164	−0.019	0.040	0.007	0.006	0.127	0.063	0.099	0.134	0.054	0.030
D13	Soft	−0.167	0.103	−0.017	0.022	−0.071	0.077	0.342	0.378	−0.020	0.121	−0.032	0.006
	Rigid	−0.298	0.117	−0.090	0.020	0.027	0.054	0.219	0.298	−0.049	0.082	0.012	0.008

## Data Availability

Not applicable.
